# Visitor Preferences for Visual Changes in Bark Beetle-Impacted Forest Recreation Settings in the United States and Germany

**DOI:** 10.1007/s00267-017-0975-4

**Published:** 2017-12-22

**Authors:** Arne Arnberger, Martin Ebenberger, Ingrid E. Schneider, Stuart Cottrell, Alexander C. Schlueter, Eick von Ruschkowski, Robert C. Venette, Stephanie A. Snyder, Paul H. Gobster

**Affiliations:** 1 Institute of Landscape Development, Recreation and Conservation Planning, University of Natural Resources and Life Sciences, Vienna, Austria; 2 University of Minnesota, Minneapolis-St Paul, USA; 3 Human Dimensions of Natural Resources, Colorado State University, Fort Collins, USA; 4 Alfred Toepfer Academy for Nature Conservation, Schneverdingen, Germany; 5 USDA Forest Service, Northern Research Station, St. Paul, MN USA

**Keywords:** Forest landscape preferences, Bark beetles, Natural processes, Cross-national comparison, Visitor numbers, Viewing distance

## Abstract

Extensive outbreaks of tree-killing insects are increasing across forests in Europe and North America due to climate change and other factors. Yet, little recent research examines visitor response to visual changes in conifer forest recreation settings resulting from forest insect infestations, how visitors weigh trade-offs between physical and social forest environment factors, or how visitor preferences might differ by nationality. This study explored forest visitor preferences with a discrete choice experiment that photographically simulated conifer forest stands with varying levels of bark beetle outbreaks, forest and visitor management practices, and visitor use levels and compositions. On-site surveys were conducted with visitors to State Forest State Park in Colorado (*n* = 200), Lake Bemidji State Park in Minnesota (*n* = 228), and Harz National Park in Germany (*n* = 208). Results revealed that the condition of the immediate forest surrounding was the most important variable influencing visitors’ landscape preferences. Visitors preferred healthy mature forest stands and disliked forests with substantial dead wood. The number of visitors was the most important social factor influencing visitor landscape preferences. Differences in the influence of physical and social factors on visual preferences existed between study sites. Findings suggest that both visual forest conditions and visitor use management are important concerns in addressing landscape preferences for beetle-impacted forest recreation areas.

## Introduction

Extensive outbreaks of tree-killing conifer forest insects such as the mountain pine beetle (*Dendroctonus ponderosae*) in North America and the spruce bark beetle (*Ips typographus*) in Europe are increasing due to climate change and other factors (Morris et al. 2015; Müller et al. 2008; Raffa et al. 2008). Mountain pine beetles have impacted more than 17 million hectares of United States (U.S.) forests since 1996 and climate change threatens to expand their impact (USDA Forest Service 2013). In Europe, the spruce bark beetle has been identified as one of its most forest destructive pests, with damage in Austria, for example, at a historic high in past years (Steyrer and Hoch 2015).

From an ecological perspective, the native bark beetle is considered a keystone species in natural forest ecosystems (Müller et al. 2008). However, extensive bark beetle outbreaks can result in timber value losses and impact non-timber values of forest owners and visitors. With respect to the latter, forest-based outdoor recreation and tourism are significant industries in North America and Europe (World Travel & Tourism Council 2016) that have the potential to be threatened by bark beetle outbreaks, resulting in loss of revenue to providers and local communities (Aukema et al. 2011; Flint et al. 2009; Rosenberger et al. 2012). Damage from insect outbreaks can also have a variety of non-economic impacts on the well-being of individuals, communities and, ultimately, society (Flint et al. 2009; Rosenberger et al. 2012).

In addition to impacts on forest recreation settings caused by beetles, social setting considerations such as visitor numbers and conflicts can influence the quality of outdoor recreation experience and constrain visitation (Manning 2011; Shelby and Heberlein 1986). The question arises of how social setting considerations are weighed by individuals relative to physical ones in beetle-impacted forests, particularly when considering limited budgets that natural resource managers may face.

Adding to our understanding of visitor responses to visual changes in beetle-impacted forest recreation settings, this study employed a discrete choice experiment (DCE) (Louviere et al. 2000) using digitally calibrated images that provided strictly controlled visual simulations to present forest stands with varying levels of beetle outbreaks, forest management practices, and visitor uses to investigate on-site forest visitors’ visual preferences. Unlike conventional univariate preference studies, this approach allows analysis of trade-offs among these forest recreation-related factors because visitors must often balance a complex set of physical and social settings in choosing among their most and least preferred recreation settings (Manning 2011).

A cross-national comparison of beetle-impacted recreational forest settings in the U.S. and Germany was conducted to gain a deeper understanding of this global issue. Previous cross-national research observed differences in preferences between the U.S. and Central European forest visitors with respect to mountainous landscapes (Rom et al. 2013). Similarly, research on crowding perceptions of Central European and Japanese forest visitors (Arnberger et al. 2010) and of USA, British and Turkish national park visitors (Sayan et al. 2013) found higher tolerances for visitor numbers among the Japanese and Turkish samples. However, it is largely unknown whether such differences in preferences for beetle-impacted landscapes and visitor numbers exist for German and U.S. forest visitors and whether these groups weigh trade-offs between physical and social forest attributes differently.

This study is conceptually rooted in the psychophysical approach to landscape preference assessment (Daniel and Boster 1976; Zube et al. 1982), in socio-psychological theories of leisure dealing with crowding and user conflict (Jacob and Schreyer 1980; Shelby and Heberlein 1986), and in the random utility theory as the basis for DCEs (McFadden 1974). The pertinent literature for each is discussed in the sections below.

### Esthetic Preferences for Forests and Forest Management

An extensive body of research on landscape esthetic preferences for conifer forests and insect-impacted forests provides solid guidance upon which to base this inquiry. Preference studies on insect-impacted coniferous forests consistently show high public sensitivity to beetle activity (Buhyoff et al. 1986; Buhyoff and Leuschner 1978; Sheppard and Picard 2006). With respect to vitality, the greener and thriving a forest appears, the more it tends to be appreciated. Conversely, the presence of dead and dying material (e.g., dead trees, logging residues) can negatively affect preferences regardless of their genesis (Buhyoff and Leuschner 1978; Buhyoff et al. 1986; Edwards et al. 2012).

Foliage color is an important indicator of beetle damage. As pine beetles infest trees, they change the trees’ appearance from green to yellow and red and eventually to gray/black, whereas spruce bark beetles change the trees’ appearance from green to gray. In coniferous forests the color of dying needles negatively influences people’s landscape preferences (Kaufman and Lohr 2008; Young and Wesner 2003). These esthetic impacts are especially felt when beetle damage is observed at near-view distances as compared to midground or background distance zones (Buhyoff et al. 1982).

Forest management in response to bark beetle infestation can differ depending on forest management goals and public forest and nature conservation policies. In core zones of protected areas, a non-intervention policy is often followed to promote natural processes and natural rejuvenation (Müller et al. 2008). However, such a policy may be in opposition to public preferences (McFarlane and Watson 2008). Outside protected areas, interventions include removal of infected and dead trees or clear cuts followed by artificial reforestation. However, clear cuts are typically disliked by forest visitors (Edwards et al. 2012; Gundersen and Frivold 2008; Ribe 1989, 1990).

A number of studies have addressed public perceptions toward the ecological and economic consequences of forest insect outbreaks (e.g., Buhyoff and Leuschner 1978; McFarlane and Watson 2008; McGrady et al. 2016; Müller et al. 2008). Yet, little is known about the influence of naturally altered conifer forest landscapes and forest management interventions and the location of the impacted forest stands (near-view to far-view) in relation to each other on forest visitors’ visual preferences (Sheppard and Picard 2006).

### Preferences for Social Settings Relating to Forest Recreation

Crowding and user conflicts influence outdoor recreation satisfaction, thus are relevant to visitor management (Jacob and Schreyer 1980; Manning 2011). Considerable research has focused on crowding perceptions, particularly in the USA where studies consistently show that high visitor numbers in natural areas reduce the quality of the recreation experience (Manning 2011; Shelby and Heberlein 1986; Vaske et al. 1996). Few equivalent European-based studies exist and comparative cross-national studies can deepen insights into these important social issues (Bakhtiari et al. 2013). Beyond numbers, user conflicts occur when the presence or behavior of individuals or groups interferes with the goals of others (Jacob and Schreyer 1980; Schneider and Hammitt 1995). Hikers, for example, are more likely to perceive conflict with bicyclists than with other hikers (Cessford 2003). The number of dogs and dog walker behavior encountered in recreational settings can also evoke conflicts with other area users, particularly when the dogs are unleashed and higher in numbers (Arnberger et al. 2010; Arnberger and Eder 2015).

Visitor management approaches range from direct exclusion of activities to more indirect methods such as informational signs that specify which activities may occur in an area or on a trail (Manning and Anderson 2012). Signs can alert visitors to expect encounters of specific activity types. Cessford (2003), for example, found that pre-informed hikers were less likely to report conflict with mountain bikers than those who were not informed about shared trail use.

While previous preference studies have integrated social aspects in their analyses, trade-offs between natural site characteristics and social settings have not been investigated in the context of bark beetle-impacted rural conifer landscapes. However, a recent study in the context of urban broadleaved forests showed that trail users in emerald-ash borer-impacted ash forests found that beetle impacts including trail-proximate EAB-related forest management responses were significant but of lesser importance than surrounding viewscape development and visitor numbers (Arnberger et al. 2017).

### Stated Preference and DCEs

The few recent preference studies that combine physical and social factors of recreation areas found recreationists integrate multiple factors in their site choices (Arnberger and Eder 2011, 2015; Bullock and Lawson 2008; Van Riper et al. 2011). Therefore, stated choice approaches seem appropriate to estimate the value of different forest conditions, management practices, and spatial and social factors within a single research design. The relative importance of social or physical aspects differs among the previously mentioned studies, yet visitor numbers and visitor behavior are found to be consistently important. Arnberger and Eder (2015), for example, showed that trail user numbers and litter were very influential in setting preferences, while Van Riper et al. (2011) found that the number of people leaving a marked trail was disliked and more important than on-trail visitor numbers and resource conditions.

Stated preference or choice approaches such as DCEs have frequently been applied to study public preferences and choice behavior concerning a range of landscape and recreation-related issues (Louviere et al. 2000; Nielsen et al. 2007; Rambonilaza and Dachary-Bernard 2007). In a DCE, two or more alternatives are combined into choice sets and respondents choose the most and/or least preferred alternative from each set they are asked to evaluate. Alternatives are defined as combinations of attributes and their levels. Random utility theory (McFadden 1974) postulates that these choices can be modeled as a function of the attributes of the alternatives. The selection of one alternative over another implies that the utility of that alternative is greater than the utility of any other alternative (Louviere et al. 2000).

### Research Questions

This study investigated recreationists’ visual preferences for forest stands with varying levels of beetle impacts, different forest management practices, and varying visitor uses. In addition, spatial aspects were integrated showing forest stands in the foreground, midground, and background of the vision field (Buhyoff et al. 1982; Sheppard and Picard 2006). This study also compared visual preferences and trade-offs between visitors to one German and two U.S. forest sites with different histories of beetle impact and management. While all sites are important tourism destinations, they differ in the degree of bark beetle infestation and visitor use densities. This study identified visitors’ preferences and tests whether trade-offs are similar across sites and countries. Study results may be useful to managers in prioritizing and tailoring their management efforts in the fields of forestry and recreation.

The following research questions guided the study:

1. What are the visual preferences of forest visitors for beetle-impacted and non-impacted forest stands and forest management strategies (intervention vs. non-intervention policy)?
2. Do preferences vary by the location of the impacted forest stands in the landscape (near-view to far-view)?
3. What trade-offs do visitors make between physical and social factors of forest recreation sites, and which attributes influence visitors’ preferences most?
4. Are there differences in landscape preferences between forest visitors to sites with different bark beetle impacts and to German and U.S. forest recreation sites?

## Methodology

### Study Sites

The study was conducted in two U.S. state parks—State Forest State Park in Colorado and Lake Bemidji State Park (LBSP) in Minnesota—and in Harz National Park (HNP) in Germany (Table 1). The sites were selected to compare public preferences for bark beetle infestations at different stages. State Forest State Park is heavily impacted by the bark beetle, resulting in many areas of infested trees, while beetle impact in the HNP is not always visible. LBSP in Minnesota is marginally affected by bark beetle infestation.

**Table 1:** Description of study sites

Site characteristics	Colorado State Forest State Park (COSP)	Lake Bemidji State Park (LBSP)	Harz National Park (HNP)
Country, federal state	USA, Colorado	USA, Minnesota	Germany, Lower Saxony
Area size	290 km^2^	7 km^2^	247 km^2^
Elevation	2500–4000 m	416 m avg.	230–1141 m
Main conifer tree species	*Pinus contorta*	*Pinus resinosa, P. strobus, P. banksiana*	*Picea abies*
Bark beetle species	Mountain pine beetle (*Dendroctonus ponderosae*)	Pine engraver beetle (*Ips pini*)	Spruce bark beetle (*Ips typographus*)
Infected trees	Many and very obvious	Few and not obvious	Many and obvious
Bark beetle management	Clear cuts near roads, utilities, and campgrounds	Selective thinning and reforestation	Clears cuts in buffer zones
Number of visitors, annually (estimated)	0.43 m	0.14 m	1.75 m
Main recreational activities	Hiking, camping, biking, wildlife viewing, OHV riding, fishing, skiing/snowboarding	Hiking, camping, wildlife viewing, cross-country skiing, snowshoeing; swimming, boating, fishing, bog walks	Hiking, mountain biking, Nordic skiing; visiting national park information center
Open for recreation use	Year round	Year round	Year round

Colorado State Forest State Park (COSP) is located in the high country of north-central Colorado, 128 km from Fort Collins. The park covers forestland west of the Medicine Bow Mountain Range and into the northern end of the Never Summer Range. Its alpine lakes, trails, and developed and backcountry sites attract visitors for a number of summer and winter recreational activities. About 95% of lodgepole pine (*Pinus contorta*), which accounts for 60% of all tree cover at the park, were killed by the mountain pine beetle (*Dendroctonus ponderosae*) (COSP 2016a). Current management of the forest for desired future conditions and forest health includes clear cuts near roads, utilities, and campgrounds (COSP 2016b).

LBSP is located in the pine moraine region of northwest Minnesota. The park affords a variety of land and water-based recreation opportunities. Coniferous species include red pine (*Pinus resinosa*), white pine (*Pinus strobus*), and jack pine (*Pinus banksiana*). A 2007–2008 infestation by pine engraver beetles (*Ips pini*) primarily impacted jack pine and red pine in smaller stands (personal correspondence, Minnesota Department of Natural Resources 2016).

HNP is located in Northern Germany and protects 10% of the Harz Mountains, which form the first major elevation inland from the North Sea coast. HNP is a major tourism destination. Forest cover is dominated by Norway spruce (*Picea abies*). Since its designation as a national park in 1990, the major objective has been to accelerate recovery of the natural vegetation through both active forest management and by natural processes. A policy of “non-management” of spruce monocultures in the core zone has led to significant spruce bark beetle outbreaks (*Ips typographus*). Bark beetles are only managed in a 500-m buffer zone from the park boundary in order to avoid spreading to neighboring private forests and along hiking trails (Nationalpark Harz 2014).

### Questionnaire

Although the questionnaire dealt with a range of topics, the focus of this paper is on items assessing respondent preferences for landscape scenes depicting beetle damage in the context of various physical and social factors. Socio-demographic and recreation behavior items, awareness of bark beetles in the area and crowding perceptions were also included for descriptive purposes. Since the project was conducted in two nations, both normative and semantic equivalence of question wording was assessed. Native researchers were part of the normative question equivalence check as well as professional back-translation to check semantic equivalence. The English questionnaire was translated into German and then back-translated by a professional translation firm.

**Fig. 1:**
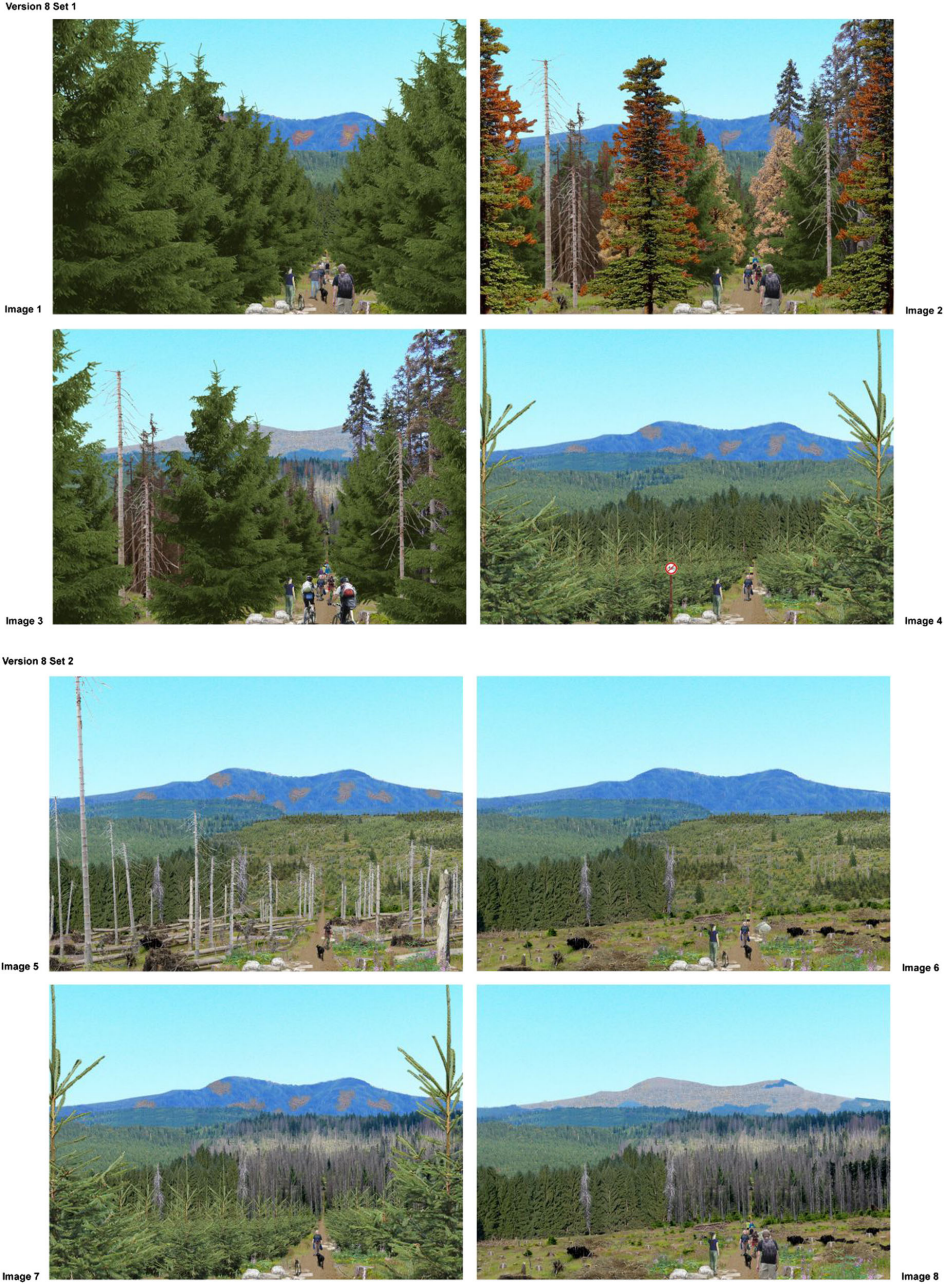

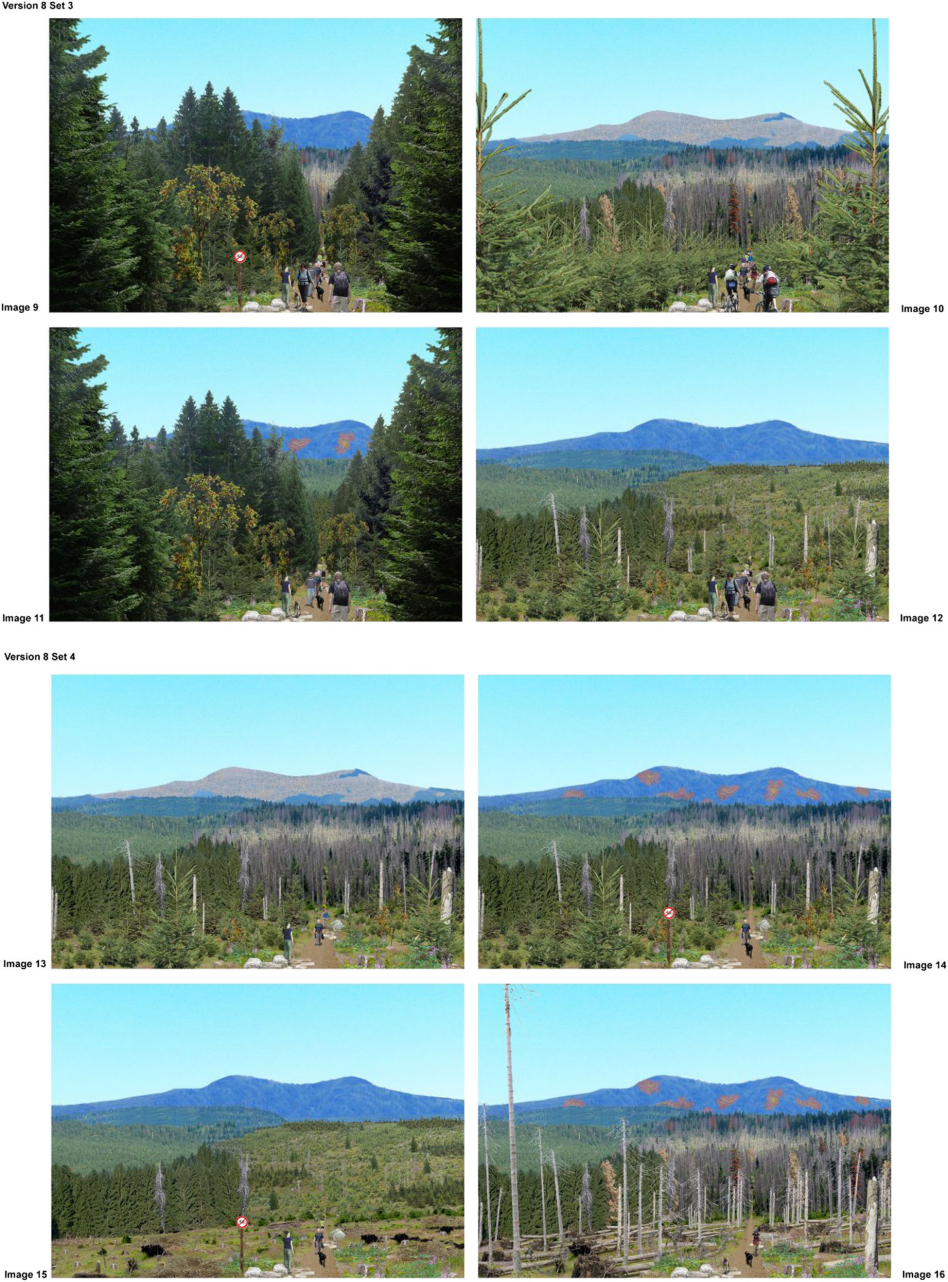
Continued

Participants evaluated alternative scenarios of forest environments displayed as photorealistic, digitally calibrated images of the DCE (Fig. 1). They chose their most and least-preferred forest environment alternative out of a choice set consisting of four images displayed on a page. In total, they evaluated four choice sets, resulting in an evaluation of 16 different forest scenarios. Respondents were not told that the photos depicted different beetle-impacted conditions and management activities.

The base photo used in all images showed a landscape of the Bavarian Forest National Park heavily affected by the spruce bark beetle, thus most of the scenarios showed spruce trees. Respondents were not shown images of the specific locale they were visiting, yet there were similarities in landscape appearance. Many landscape preference studies have successfully used images that were unfamiliar to the study respondents or different from the specific locale respondents were visiting (Arnberger et al. 2010; Rom et al. 2013; Tahvanainen et al. 2001).

The forest scenarios were developed by a team of foresters, park managers, recreation planners, and landscape architects. Each photorealistic forest scenario depicted six physical and social attributes, with multiple levels of each attribute varying systematically in combination with other attributes and levels to represent logically occurring alternatives that a visitor might encounter in the landscape (see Fig. 1, Table 3). Three physical attributes characterized forest conditions that can occur in mountain pine and spruce bark beetle-impacted landscapes along three viewing distances: immediate foreground, midground, and background. For the immediate foreground, the DCE tested eight levels of forest stand scenarios simulating a typical bark beetle outbreak, from unaffected conditions, through partial and full stages of impact, to post-impact recovery. These levels were further distinguished by two alternative forest management strategies—a non-intervention strategy featuring a scenario where dead standing and fallen trees were visible (collapse scenario), followed by natural rejuvenation and a natural forest; vs. an intervention strategy with simulated clear cuts after first partial stages of impact, artificial rejuvenation, and a commercial forest stand. The midground and background attributes each included four treatment levels ranging from non-impacted closed forest conditions, to partial and complete impact or to natural rejuvenation.

Three social attributes were used to examine preferences for intensities and types of trail uses as potential causes for crowding and visitor conflict. Scenarios displayed the number of visitors (four levels from 1–12 visitors), user groups (four levels showing varying proportions of walkers and mountain bikers), and number of dogs and dog walkers’ behavior (no dog, one dog leashed, two dogs leashed, two dogs unleashed). A visitor management measure, the presence or absence of a trail sign prohibiting bicycling, was also employed. The sign prohibiting bicycling was always combined with a 100% proportion of hikers in the scenarios.

The exact combination of the attribute levels of the DCE depended on an underlying asymmetric orthogonal fractional factorial design (Louviere et al. 2000), which required 128 scenarios. Combining scenarios to choice sets, and blocking the choice sets to 16 survey versions, also followed this statistical design plan. The forest scenarios were calibrated using Adobe Photoshop by storing all factor levels on individual layers. When compiling the 128 scenarios, the layers ensured that the provision of a specific level was always the same, independent of the other forest settings.

### Data Collection

Data were collected during summer 2014 using a structured questionnaire distributed through an on-site intercept approach. To avoid starting point bias the choice sets of the DCE were rotated systematically (Gibson et al. 2014) across successive respondents. Data collection included a stratified-cluster sample of visitors with a systematically selected sampling period that varied by time of day and day of the week to reflect each park’s visitation patterns and capture a diverse visitor segment. Researchers were stationed at frequently visited park locations such as visitor centers, boat launches, trailheads, and picnic shelters. Only adult visitors were asked to participate; if respondents completed the questionnaire and indicated they were under 18, their responses were not included in the data. No incentives were offered to respondents. Data collectors registered group size, activity type, date, and specific location. Sample size of the COSP was 200 (response rate = 65%), in LBSP it was 228 (response rate = 74%), and in HNP it was 208 (response rate = 49%). Reasons for non-response were either time-related—visitors did not want to disrupt their activity—or a lack of interest in participating in the study. For HNP, refusal rates per activity type were collected. Most of non-respondents were hikers/walkers (93.6%), followed by dog walkers (4.5%) and bicyclists, joggers and Nordic walkers (1.9%). The lower response rate may have resulted in a non-response bias. However, many studies have found a weak relationship between response rates and non-response bias (Johnston et al. 2017).

### Data Analyses

Chi-square and ANOVA tested for differences in socio-demographic and visit-related variables among the samples. All attribute levels of the DCE were effects coded, where an N-categorical variable is defined by N-1 estimates only (Louviere et al. 2000). Since the model was designed as a multivariate study with six variables, the multinomial logit model estimates were all relative to each other. No base alternative or “no-choice” alternatives were presented. Therefore, no intercept exists. The maximum likelihood analysis produces parameter estimates (part-worth utilities), *z*-values and standard errors for each attribute level. McFadden’s *ρ* ^2^ was used to indicate the goodness of fit of the estimated choice models, analogous to *R* ^2^ in ordinary regression. Values of *ρ* ^2^ between 0.2 and 0.4 indicate extremely good model fits (Louviere et al. 2000). The DCE analyses resulted in very reliable models with *ρ* ^2^ statistics ranging from 0.26 to 0.31 (Table 3). A Wald statistic tested differences between the samples. Latent Gold Choice 4.5 statistical software was used for modeling (Vermunt and Magidson 2003). The relative importance of each attribute on landscape preferences was calculated following Vermunt and Magidson (2003). The more positive the parameter estimate of an attribute level, the more preferred among the sample.

## Results

### Sample Characteristics

Visitors to the HNP were older and more often rode a bike within the last 12 months than visitors to the U.S. state parks (Table 2). No differences among the samples existed for gender. Differences in the main purposes of the visit were found across the sites. Hiking/walking was the main purpose of HNP respondents, while camping was the main one for the other samples. On average, COSP respondents made their first area visit in 2005, LBSP respondents in 2000, and HNP respondents in 1986. Dog ownership was highest in COSP followed by LBSP and HNP. Crowding perceptions were highest in COSP and lowest in HNP and indicated that respondents felt slightly crowded on average. Finally, LBSP visitors were much less aware of bark beetles in the area (21%) compared to the HNP sample (69%) and, in particular, to the COSP sample (95%).

**Table 2:** Socio-demographics and forest visit-related variables

Variables	COSP (*n* = 200)	LBSP (*n* = 228)	HNP (*n* = 208)	Differences ANOVA/*χ* ^2^
Age (years, mean)	45.9	44.8	48.2	*F* = 3.042*
Gender (females)	41%	49%	48%	*χ* ^2^ = 3.676
Main purpose of visit				
Hiking/walking	23%	14%	69%	*χ* ^2^ = 301.313***
Camping	35%	30%	0%	
Relaxing	15%	19%	6%	
Landscape/nature observation/photography	1%	2%	16%	
Bicycling	1%	14%	2%	
Fishing	15%	3%	0%	
Others (swimming, running, hunting, OHV-riding …)	11%	18%	7%	
First area visit ever (year, mean)	2005	2000	1986	*F* = 76.077***
Awareness of infestation of the visited area				
Yes	94%	21%	69%	*χ* ^2^ = 254.784***
No	1%	5%	6%	
Do not know	6%	74%	25%	
Crowding perceptions (mean)^a^	3.7	3.5	3.1	*F* = 4.771**
Dog ownership				*χ* ^2^ = 92.518***
Yes	62%	47%	18%	
No	32%	45%	59%	
Used to have a dog	6%	8%	23%	
Number of times ridden a bike within the last 12 months (mean)	41.7	32.2	121.2	*F* = 32.687***

*COSP* Colorado State Forest State Park, CO, *LBSP* Lake Bemidji State Park, MN, *HNP* Harz National Park, Germany

*p < .05

**p < .01

***p < .001

^a^ Perceived area crowding from 1 = “not at all crowded” to 9 = “extremely crowded”

### Preferences for Physical Forest Factors

**Fig. 2:**
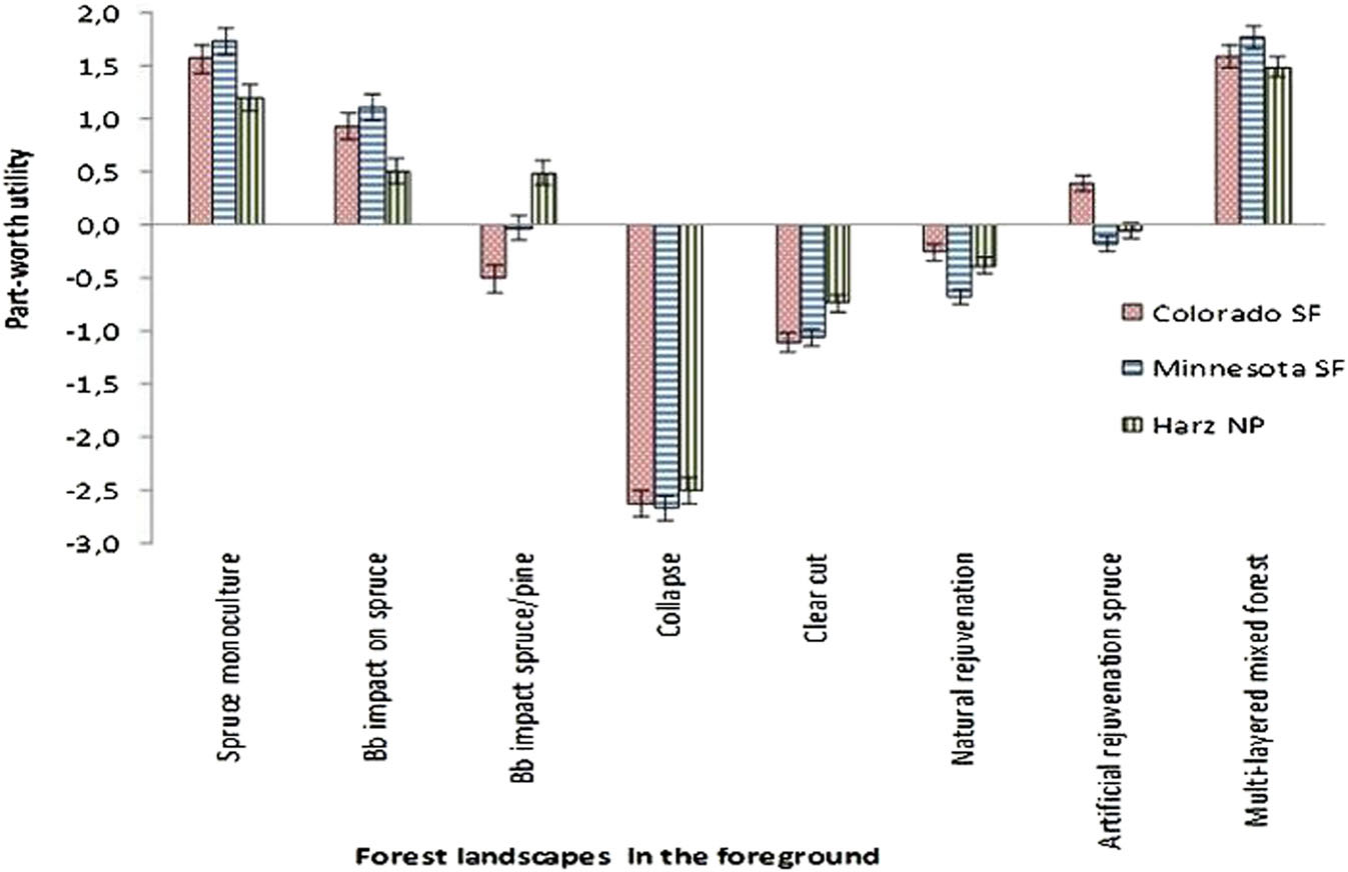
Part-worth utility parameter estimates for the attribute levels “forest foreground”. Bb bark beetle, SF State Forest, NP National Park

For the foreground attribute, respondents preferred mixed and multilayered forest stands and monocultures of mature spruce trees without any beetle impacts (Table 3, Fig. 2). The spruce scenario portraying the beginning of a bark beetle infestation, with some light openings and patches and low amounts of dead standing trees, received lower positive evaluations in each study site than a mature forest, but evaluations were more positive than for stages where the forest is largely brown and dead. The lowest value of this attribute was found for the collapse scenario where all trees were dead, followed by a clear cut with the removal of dead wood leaving some visible traces of human intervention. For the rejuvenation treatment levels, respondents preferred artificial reforestation with young spruce trees of the same age class over a natural succession of mixed pine and spruce. HNP visitors showed a higher preference for pine/spruce beetle-impacted trees compared to beetle-impacted spruce monocultures, while COSP visitors showed opposite preference patterns.

**Table 3:** Parameter estimates and Wald statistics for attributes and attribute levels

	COSP	LBSP	HNP		Differences among samples
Attributes and attribute levels	Parameter estimates	Parameter estimates	Parameter estimates	Wald statistic	
Forest landscape—foreground					
Spruce monoculture	***1.571	***1.738	***1.199	***101.85	COSP≠LBSP≠
Bark beetle impact on spruce only	***0.938	***1.109	***0.506		HNP ≠ COSP
Bark beetle impact spruce/pine mixed	***−0.503	−0.027	***0.488		
Collapse—only dead wood	***−2.632	***−2.670	***−2.504		
Clear cut with logging traces	***−1.108	***−1.054	***−0.740		
Natural rejuvenation mixed	**−0.256	***−0.680	***−0.384		
Artificial rejuvenation spruce	***0.396	**−0.184	−0.055		
Multi-layered mixed forest	***1.594	***1.767	***1.490		
Forest landscape—midground					
Non-impacted, closed forest	***0.418	***0.578	***0.630	*14.10	COSP≠HNP
Bark beetle impact on spruce	−0.084	**−0.177	***−0.371		
Bark beetle impact spruce/pine mixed	***−0.290	***−0.347	***−0.279		
Natural rejuvenation mixed	−0.044	−0.055	0.019		
Forest landscape—background					
Non-impacted, closed forest	0.000	***0.239	**0.155	*13.60	COSP≠LBSP
Bark beetle impact on spruce only	0.106	−0.083	0.071		
Bark beetle impact spruce/pine mixed	*−0.125	−0.058	**−0.173		
Collapse—only dead wood	0.019	−0.098	−0.052		
Dog walker behavior					
No dog	0.077	−0.015	*0.157	3.83	
1 dog leashed	−0.039	0.003	−0.056		
2 dogs leashed	−0.089	−0.043	*−0.129		
2 dogs unleashed	0.015	0.061	0.027		
User composition					
100% walkers, no cyclists—prohibition sign	−0.042	***−0.270	*−0.129	8.95	
75% walkers, 25% cyclist—cycling allowed	0.048	**0.144	0.036		
25% walkers, 75% cyclists—cycling allowed	*−0.123	−0.054	−0.019		
100% walkers, no cyclists—cycling allowed	0.118	**0.179	0.111		
Number of visitors					
1 Person	***0.346	*0.128	**0.159	***48.02	COSP≠LBSP≠
4 Persons	***0.534	***0.230	***0.544		HNP≠COSP
8 Persons	**−0.228	***−0.230	***−0.308		
12 Persons	***−0.652	*−0.126	***−0.396		
*ρ* ^2^	0.313	0.302	0.256		

*COSP* Colorado State Forest State Park, CO, *LBSP* Lake Bemidji State Park, MN, *HNP* Harz National Park, Germany

Significant influence of the attribute levels on respondents’ choices (*N* = 636): *p < .05; **p < .01; ***p < .001

COSP≠LBSP≠HNP≠COSP: Significant differences between all study sites using pairwise comparisons at least at the *p* < .05 level

**Fig. 3:**
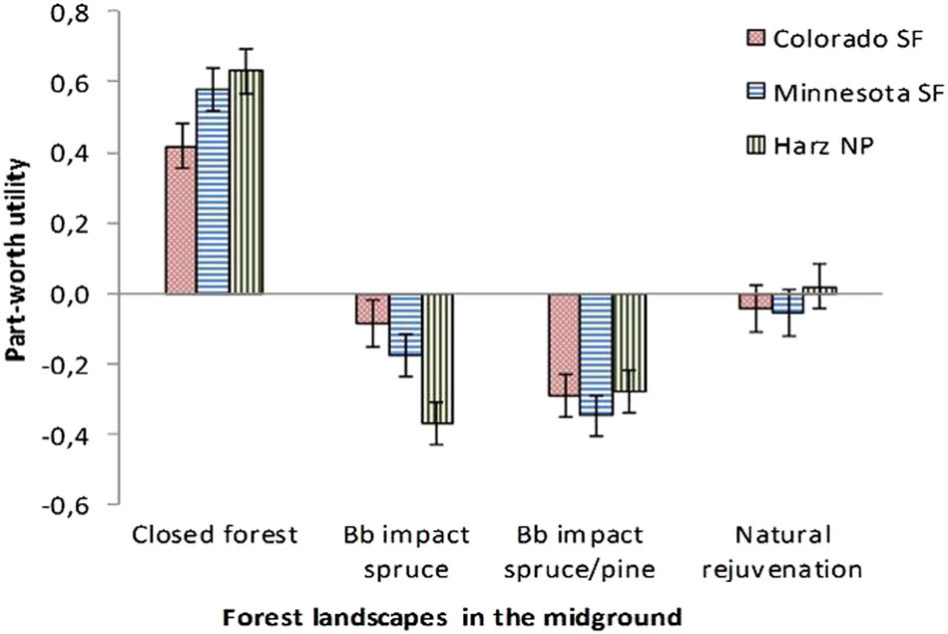
Part-worth utility parameter estimates for the attribute levels “forest midground”. Bb bark beetle, SF State Forest, NP National Park

For the midground attribute, visitors at all sites preferred a closed, mature, and unaffected forest stand, whereas they disliked scenarios depicting yellow-colored forest patches, which indicated pine beetle impacts (Fig. 3). HNP visitors evaluated the bark beetle impact of pure spruce stands more negatively than COSP visitors. For the background attribute, visitors preferred a closed forest and disliked bark beetle-impacted mixed pine and spruce forest stands. LBSP visitors showed a higher preference for unaffected forests compared to COSP visitors.

### Preferences for Social Factors

**Fig. 4:**
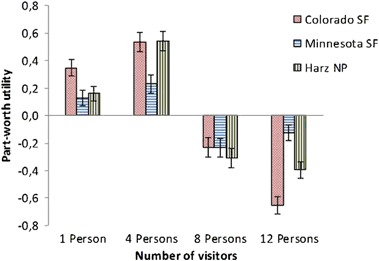
Part-worth utility parameter estimates for the attribute levels “number of visitors”. SF State Forest, NP National Park

In scenarios where the number of visitors exceeded four persons, preference for the forest recreation site decreased, particularly for the COSP and HNP samples (Fig. 4). Scenarios with hikers and without many bikers received the highest parameter values if cycling is allowed. LBSP and HNP visitors disliked the combination of a trail sign, which prohibits bicycling, with a 100% proportion of hikers. Dog walker behavior was the only attribute without significant influence on participants’ choices except for HNP visitors, who preferred no dogs in the landscape and disliked two dogs on a leash (Table 3).

### Relative Importance of the Attributes

In relation to the other attributes, the appearance of the near-view forest surroundings was by far the most important predictor for recreationists’ landscape preferences, followed by the appearance of forests located in the midground landscape and the number of visitors (Table 4). The forest environment in the background was rather unimportant to participants. The number of visitors was the most important social factor influencing visitor landscape preferences followed by visitor composition. For COSP respondents, the number of visitors was more important than the forest environment in the midground, whereas at LBSP visitor numbers were less important than the midground landscape. Non-significant variations in relative importance were found among the study sites for the other attributes.

**Table 4:** Relative importance of attributes for the choices of each sample

Attributes	COSP	LBSP	HNP
Forest landscape—foreground	62.5%	66.1%	58.8%
Forest landscape—midground	10.5%	13.8%	14.7%
Forest landscape—background	3.4%	5.0%	4.8%
Dog walker behavior	2.5%	1.6%	4.2%
User composition	3.6%	6.7%	3.5%
Number of visitors	17.6%	6.9%	13.8%

*COSP* Colorado State Forest State Park, CO, *LBSP* Lake Bemidji State Park, MN, *HNP* Harz National Park, Germany

## Discussion

This study adopted an image-based choice experiment approach to investigate a wide variety of conifer forest landscapes and visitor uses recreationists typically encounter in bark beetle-impacted forests. This paper integrated physical, managerial, and social aspects and distance effects in one study design to systematically compare public preferences across three sites with different stages of bark beetle impact. Results revealed that the condition of the immediate forest surrounding was the most important variable and that preferences for forest management practices varied. Differences in the influence of physical, managerial, and social factors on visual preferences existed between study sites.

### Preferences and Trade-Offs for Forest Site Characteristics and Management

Supporting past research results, this study found that the appearance of the forest environment with its natural and managed components, as well as the number of visitors, most influenced participants’ landscape preferences. Respondents preferred to see a forest in a climax stand with large, green trees and a (nearly) closed canopy (Edwards et al. 2012; Gundersen and Frivold 2008; Ribe 1989, 1990). Also similar to past research (Buhyoff and Leuschner 1978; Buhyoff et al. 1982, 1986; Sheppard and Picard 2006), respondents disliked severe bark beetle outbreaks with high amounts of dead wood and clear cuts. Analyses of spatial aspects of forest insect impacts showed that with increasing distance, the appearance of the landscape had a decreasing influence on respondents’ preferences (Buhyoff et al. 1982). As such, the focus of visual resource management in bark beetle-impacted landscapes should be on the forest immediately surrounding hiking trails and tourism facilities. Clear cuts along trails should be avoided as long as remaining trees pose no threat to visitor safety. Selective removal of standing dead and dying trees and removal or relocation of logging residues away from trails, if not contradicting management goals, may be another option to maintain the recreation experience of visitors. In some cases, there may options for temporarily closing or diverting trails away from impacted areas until new forest growth is established, though such options are often limited where there are widespread impacts or limited rerouting alternatives.

Respondents expressed a slightly higher preference for an intervention strategy over natural rejuvenation. Specifically, a clear cut followed by an artificial reforestation with a monoculture of even-aged spruce trees was preferred to a dead wood (collapse) scenario followed by a natural rejuvenation. The higher preference for an intervention strategy found in this study may pose a challenge for natural management of protected areas if management restrictions exist. Management could intensify educational efforts about bark beetles and their role in ecological processes and dynamics of forest ecosystems to explore whether this could increase the acceptance of successional stages with high amounts of dead trees. An effective communication strategy to address the associated issues could be the provision of information along hiking trails and around tourism facilities in and near impacted forest stands. So far, limited research exists on the influence of knowledge on visitor perceptions of bark beetle-impacted forests (McFarlane and Watson 2008; McGrady et al. 2016; Müller et al. 2008) and little research has examined knowledge of pest-specific information on landscape preferences (Buhyoff et al. 1982). Schlueter and Schneider (2016) found a significant, weak positive relationship between accepting two management approaches and beetle knowledge. Though some research takes into account the context of invasive species, few reports assess forest-pest knowledge and its impact on landscape preference (Buhyoff et al. 1982; Ryan 2012). Respondents in our study were not told that the pictures they were examining contained beetle damage, but they were asked about their awareness of infestation in the area they were visiting. It is possible that the greater familiarity and awareness of pest infestations among COSP and HNP respondents implicitly influenced some of the landscape preference rankings. However, our results tend to indicate that general levels of awareness about pest infestations did not have a great impact in landscape preferences. An important area of future research would be to examine the influence of knowledge or experience with forests pests on landscape preferences.

When physical and social factors were integrated into the single design, study findings differed from other studies in that social factors were rather unimportant compared to most of the physical ones. Thus, these trade-offs show that desirable social factors have little ability to moderate undesirable physical site attributes. Past research found visitor numbers played a more important role in trail preferences than our study found (Arnberger et al. 2017; Arnberger and Eder 2011, 2015; Bullock and Lawson 2008; Van Riper et al. 2011). One possible explanation is that compared to studies which focused on near-view trail conditions, this study had a landscape-scale perspective. Consequently, the magnitude of beetle impacts as depicted in the foreground and midground dominated the field of view compared to the depictions of visitors and dogs. Nevertheless, the photos depicted were realistic and based on an actual photo and therefore present current or future forest conditions. A second possible explanation is that visitors for whom the number of people or dogs matter have already been displaced (Manning 2011; Schneider et al. 2011).

Results related to the social factors confirm two key findings from past research: (1) participants dislike a high number of trail users (Arnberger et al. 2010; Arnberger and Eder 2015; Bakhtiari et al. 2013; Bullock and Lawson 2008; Manning 2011; Shelby and Heberlein 1986; Van Riper et al. 2011; Vaske et al. 1996) and (2) visitor numbers are more important than visitor composition and behavior (Arnberger et al. 2010; Arnberger and Eder 2015; Shelby and Heberlein 1986). Management options to address visitor density ranges from setting visitor expectations about how many people you might expect to see in a site to limiting use through site design, fees or permits (Manning and Anderson 2012). However, forest managers should be aware that the relative importance of social attributes is low compared to forest attributes.

Multiple-use trails invoke a number of management opportunities and challenges. Previous visitor surveys reveal user conflicts between mountain bikers and hikers can impact the recreation experience (Cessford 2003; Jacob and Schreyer 1980; Schneider and Hammitt 1995; Watson et al. 1991) as can dog walkers (Bakhtiari et al. 2013). In line with the literature is the expressed dislike of many bicyclists in the scenes. However, the visitor management attribute examined in this study—a trail sign prohibiting bicycling—was not preferred and respondents favored shared trails with little bike use. As questions specifically focused on the management options were not asked, it is difficult to interpret whether this dislike is based on the presence or type of sign or because respondents like bicycling since most of them cycled within the past year. In addition, cycling was allowed on designated trails in all sites.

### Differences among Study Sites

Two explanations for the differences among study site results are possible: (1) national differences and (2) social site conditions. A near-view forest stand with the same structures but showing pines with yellow tree foliage was less preferred in the U.S. study sites, especially in Colorado, compared to German visitors, while German visitors were more concerned about spruce beetle impacts. Previous studies have shown that the color of foliage has an influence on people’s preferences for trees and forests (Buhyoff et al. 1982; Kaufman and Lohr 2008; Young and Wesner 2003), as this study confirms cross-nationally. German respondents showed less dislike of pine beetle impacts, probably because this effect is rather unknown in Germany as needles of affected spruce trees do not turn into yellow-red colors. The same issue may have influenced responses from U.S. respondents, who evaluated the bark beetle impact of pure spruce stands more positively than HNP visitors. Beside these tree-specific differences between the countries, differences in preferences for physical forest characteristics were marginal, although the bark beetle differently impacts the study sites. This could be an indication of universal landscape preferences.

Previous research on recreational forests is limited on whether and how visitor preferences for social conditions might differ nationally or internationally. Similar to this study, existing research reveals differences in preferred social conditions between forest visitors from different countries (Arnberger et al. 2010; Sayan et al. 2013). While differences for visitor numbers emerged across all three sites, the highest differences existed between COSP and LBSP samples, both in the U.S. These study sites differ in recreation use conditions with highest visitor density in the LBSP, and lowest in COSP. However, crowding perceptions were lowest in LBSP and highest in COSP. In accordance with perceptions of crowding, LBSP visitors assigned visitor numbers the lowest relative importance in contrast to COSP and HNP visitors. This indicates that the LBSP group have the highest tolerance toward visitor numbers among all sites. In contrast, COSP visitors tolerate higher bark beetle impacts on the forest landscape when visitor use levels are low because the number of visitors was their second most important attribute. The rather remote COSP attracted visitors with preferences for low use levels. Attributes such as wilderness and solitude are probably more prevalent among the visitors to COSP than LBSP or Harz.

Dogs and dog walker behavior played no or a marginal role in this study; only HNP visitors disliked the presence of dogs. Compared to other studies in the urban context (Arnberger et al. 2010, 2017; Arnberger and Eder 2015; Bakhtiari et al. 2013), this result was a bit surprising. Perhaps the high proportion of dog owners (current and previous) or dog walkers among the U.S. samples led to their neutral evaluation of dogs and dog walker behavior. Future research could include interviews with respondents or surveys focused more specifically on the impact, or lack thereof, of dogs in these settings.

As such, it may be that the study site with its specific visitor structure is more relevant than nationality in terms of preferences for or attitudes toward social attributes. Given that none of our study sites were urban, future research may compare preferences of forest visitors in one country along a gradient from urban to rural, to examine whether degree of urbanization/ruralness may influence preferences for recreation site conditions.

## Conclusions

This study found that physical and social attributes of bark beetle-impacted forests influenced visitors’ preferences, with the condition of the immediate forest surrounding being the most influential. We suggest that forest managers and planners need to be aware of how forest insect impacts can affect recreation setting preferences as increasing outbreaks of forest insects may occur due to climate change and global trade. If forest recreation sites are heavily impacted by forest insects, then their attractiveness will diminish and visitors may avoid visiting such forest environments, leading to reductions in tourism revenues and loss of other cultural ecosystem services, as well as the potential for crowding or conflict at other recreational sites if users shift locations.

Based on this study, forest recreation managers in areas with beetle impacts have several options to maintain the quality of recreation experience for their visitors. Management, remediation, or intervention activities that focus on the forest setting proximate to the recreation areas may have high potential to address visitor perceptions and preferences. Several studies support the notion that to provide information of the issues to visitors increases their interests and understanding of what they see. This is especially true in protected areas and in places where outbreaks are natural disturbance regimes in forests. It is important to understand factors that influence changes to public perception of bark beetle outbreaks. Landscape esthetics are of particular concern among visitors as landscape appearance influences visitor experience and the frequency of subsequent visitation (Sheppard and Picard 2006). Viewing and experiencing high-quality landscapes are significant motivations for outdoor recreation. As such, esthetics and perceptions of the “natural” environment are significantly important, yet remain relativity unquantified in the context of bark beetle outbreaks. Future research could focus on whether landscape preferences might change after respondents are made aware of the source of landscape disturbance.
